# Bone and bone derived factors in kidney disease

**DOI:** 10.3389/fphys.2024.1356069

**Published:** 2024-03-01

**Authors:** Petra Simic

**Affiliations:** ^1^ Division of Nephrology, Massachusetts General Hospital, Boston, MA, United States; ^2^ Endocrine Unit, Massachusetts General Hospital, Boston, MA, United States

**Keywords:** renal osteodystrophy, CKD-MBD, mineral and bone disorder, CKD, FGF23 = fibroblast growth factor 23

## Abstract

**Purpose of review:** Mineral and bone disorder (MBD) is a prevalent complication in chronic kidney disease (CKD), significantly impacting overall health with multifaceted implications including fractures, cardiovascular events, and mortality. Despite its pervasive nature, effective treatments for CKD-MBD are lacking, emphasizing the urgency to advance understanding and therapeutic interventions. Bone metabolism intricacies, influenced by factors like 1,25 dihydroxy vitamin D, parathyroid hormone (PTH), and fibroblast growth factor 23 (FGF23), along with intrinsic osseous mechanisms, play pivotal roles in CKD. Skeletal abnormalities precede hormonal changes, persisting even with normalized systemic mineral parameters, necessitating a comprehensive approach to address both aspects.

**Recent findings:** In this review, we explore novel pathways involved in the regulation of systemic mineral bone disease factors, specifically examining anemia, inflammation, and metabolic pathways. Special emphasis is placed on internal bone mechanisms, such as hepatocyte nuclear factor 4α, transforming growth factor-β1, and sclerostin, which play crucial roles in the progression of renal osteodystrophy.

**Summary:** Despite advancements, effective treatments addressing CKD-MBD morbidity and mortality are lacking, necessitating ongoing research for novel therapeutic targets.

## Introduction

Mineral and bone disorder (MBD) represents a prevalent complication among patients with chronic kidney disease (CKD), exerting a significant impact on their overall health and wellbeing. The ramifications of CKD-associated MBD are multifaceted, extending to adverse clinical outcomes such as fractures, cardiovascular events, and, in severe cases, mortality (Martinez-Calle et al., 2023). This complex condition encompasses various manifestations, including systemic mineral metabolism disorder, renal osteodystrophy, and vascular or soft tissue calcification (Kidney Disease: Improving Global Outcomes CKDMBDUWG, 2011), all of which contribute to the heightened risk of cardiovascular events (Block et al., 2004; Moe, 2006; Isakova et al., 2011).

Regrettably, despite the pervasive nature of CKD-MBD, there is currently a lack of effective treatments for this condition, underscoring the urgency and significance of advancing our understanding of disease and therapeutic interventions. The intricacies of bone metabolism, both in the baseline state and within the context of CKD, are governed by a delicate interplay of bone mineral factors. Circulating elements such as 1,25 dihydroxy vitamin D, parathyroid hormone (PTH), and fibroblast growth factor 23 (FGF23) exert their influence on bone health, while intrinsic osseous mechanisms also play a pivotal role (Wesseling-Perry et al., 2012; Pereira et al., 2021).

Notably, the importance of intrinsic mechanisms becomes evident as skeletal abnormalities precede hormonal changes in CKD (Schiavi and Moyses, 2016), emphasizing the need to delve into the nuanced interplay between these factors. Furthermore, it is noteworthy that these skeletal irregularities persist even when systemic mineral parameters are normalized, adding another layer of complexity to the management of CKD-associated MBD (Pereira et al., 2009; Pereira et al., 2019). Consequently, a comprehensive approach to understanding and addressing both hormonal and intrinsic factors is imperative for the development of effective therapeutic strategies aimed at ameliorating the burden of CKD-MBD and mitigating its associated clinical consequences.

## Systemic mineral disorder in kidney disease

Systemic mineral disorder in CKD encompasses a complex interplay of elevated phosphate, FGF23, and PTH, along with reduced 1,25 dihydroxy vitamin D and calcium levels. Current management strategies primarily focus on normalizing systemic bone mineral parameters. The elevated phosphate levels are addressed through dietary phosphate restriction and the use of phosphate binders. Calcium imbalances are managed with vitamin D supplementation and analogs. Elevated PTH levels are controlled by regulating phosphate and calcium levels, administering vitamin D therapy, and occasionally employing medications directly targeting PTH levels. However, these treatments have not demonstrated significant improvements in the morbidity or mortality associated with CKD-associated MBD.

MBD is believed to be triggered by phosphate retention and the rise in FGF23 levels (Isakova et al., 2011). Elevated serum phosphate has been linked to cardiovascular morbidity and mortality in kidney injury patients, with vascular and coronary artery calcification identified as potential links between abnormal mineral metabolism, particularly hyperphosphatemia, and cardiovascular events in this population (Block et al., 2004; Moe, 2006; Isakova et al., 2011). Notably, serum phosphate levels often remain within the normal range in early and intermediate stages of CKD due to compensatory increases in FGF23 and PTH.

FGF23 stands out as a pivotal factor in the context of CKD, demonstrating its significance by being the earliest measurable MBD factor to increase, surpassing alterations in phosphate or PTH levels (Isakova et al., 2011). The clinical relevance of FGF23 extends beyond its early detection, as its levels are strongly correlated with the progression of kidney disease, left ventricular hypertrophy, vascular disease, and mortality. Notably, FGF23’s predictive value exceeds that of serum phosphate levels, particularly in patients with ostensibly normal serum phosphate levels (Gutierrez et al., 2008; Gutierrez et al., 2009).

The intricate mechanisms underlying the elevation of FGF23 in CKD are not yet fully elucidated. However, emerging evidence suggests that inflammation and disruptions in iron homeostasis may act as contributing factors (David et al., 2017).

Addressing anemia-related factors in CKD has unveiled potential avenues for modulating FGF23 levels. Interventions such as erythropoiesis-stimulating agents and hypoxia-inducible factor prolyl hydroxylase inhibitors have shown promise in attenuating elevated FGF23 levels in CKD, with the added benefit of potentially reducing renal fibrosis markers (Noonan et al., 2020). Roxadustat, a novel hypoxia-inducible factor stabilizer primarily used to treat anemia, has demonstrated notable effects in CKD. In rats with CKD, roxadustat not only ameliorated renal anemia but also mitigated the excessive increase in PTH and FGF23. Further bone histomorphometric analysis revealed that roxadustat significantly alleviated bone loss and deterioration in bone microarchitecture by enhancing osteoblast activity and inhibiting osteoclast activity (Li et al., 2023).

In the realm of inflammatory pathways implicated in CKD, various cytokines have been identified as contributors to elevated FGF23 levels. For example, interleukin-6 has been shown to play a role in promoting high FGF23 levels in CKD models. Additionally, interleukin-1β induction has been linked to increased FGF23, and the use of a neutralizing antibody to interleukin-1β effectively blocked FGF23 expression in both congenital CKD models and nephrotoxic serum-mediated models. Furthermore, tumor necrosis factor has been identified as a factor contributing to elevated plasma FGF23 levels, inducing ectopic renal *Fgf23* expression (Durlacher-Betzer et al., 2018; Egli-Spichtig et al., 2019; McKnight et al., 2020).

In contrast to other MBD-related hormones, FGF23 exhibits elevation not only in CKD but also in acute kidney injury (AKI). Notably, higher FGF23 levels preceding cardiac surgery or upon admission to the intensive care unit have been found to correlate with the subsequent development of AKI. Once AKI is established, increased FGF23 levels are associated with the need for renal replacement therapy and heightened mortality rates (Leaf et al., 2017; Christov et al., 2019). Paralleling the scenario observed in CKD, the elevation of FGF23 in AKI has been demonstrated to be mediated by factors such as anemia, inflammation, and metabolic pathways.

Recent research has unveiled the involvement of the glycolysis intermediate, glycerol-3-phosphate (G3P), in upregulating FGF23 in the context of AKI and in response to phosphate load (Simic et al., 2020; Zhou et al., 2023). In the milieu of AKI, G3P is secreted from the kidney in both mice and humans. It circulates to reach the bone and bone marrow, where it undergoes a transition to lysophosphatidic acid through the enzyme glycerol-3-phosphate acyltransferase (76). Subsequently, lysophosphatidic acid signals through lysophosphatidic receptor type 1 to recruit vitamin D receptor to *Fgf23* promotor and augment the transcription of *Fgf23* and concurrently decrease serum phosphate levels (Simic et al., 2020).

The liver has also been implicated in the pathogenesis of AKI-associated FGF23 elevation. IL-6 has been identified as a key player, as it increases FGF23 transcription and production during AKI. IL-6 serves as a regulator of the orphan nuclear receptor estrogen related receptor γ (ERR-γ). Administration of an IL-6 antibody in a folic acid-induced AKI model attenuated the effects on ERR-γ and FGF23 production in the liver (Radhakrishnan et al., 2021). Additionally, AKI patients undergoing cardiac surgery exhibited a significant correlation between elevated IL-6 and FGF23 levels, providing predictive value for the occurrence of AKI (Radhakrishnan et al., 2021).

The role of erythropoietin, a known regulator of FGF23, has garnered attention in the pathogenesis of AKI. Blocking the erythropoietin receptor prevented the induction of bone marrow FGF23 and reduced the increase in plasma FGF23 in hemorrhagic shock or sepsis-induced AKI rodent models (Toro et al., 2018).

Despite the promising insights into the role of FGF23 in the context of CKD, direct targeting of FGF23 for therapeutic purposes presents a complex challenge. Experimental studies involving the neutralization of FGF23 in CKD animal models have yielded mixed outcomes, demonstrating both positive and adverse effects on various biochemical parameters. In these studies, neutralizing FGF23 resulted in a decrease in PTH levels, an increase in vitamin D and calcium concentrations, and the normalization of bone markers. While these effects might seem promising for addressing CKD-MBD, they were accompanied by adverse consequences (Shalhoub et al., 2012). Contrary to the anticipated benefits of FGF23 neutralization, the intervention led to an undesirable increase in circulating phosphate levels. Elevated serum phosphate is a critical factor associated with the progression of CKD-MBD and contributes to the development of aortic calcification and cardiovascular complications (Shalhoub et al., 2012). Most significantly, the CKD-MBD rats treated with the FGF23 antibody had increased mortality. While FGF23 has been recognized as a key player in mineral metabolism regulation, these findings emphasize the potential unintended consequences of altering its levels directly.

In the realm of PTH dynamics in CKD, the renal actions that normally promote the synthesis of 1,25-vitamin D become compromised, even in the presence of secondary hyperparathyroidism. This compromised synthesis is attributed, in part, to the downregulation of PTH receptors within the damaged kidney, which contributes to a reduction in the renal production of 1,25-vitamin D. In efforts to address these challenges, there has been a proposal to target downstream signaling steps of PTH using salt-inducible kinase inhibitors (Yoon et al., 2023). This approach aims to stimulate 1,25-vitamin D synthesis in a podocyte injury CKD-MBD model, potentially circumventing the defects arising from PTH receptor downregulation and elevated FGF23 levels (Yoon et al., 2023). Additionally, salt-inducible kinase inhibitors directly increase bone formation and bone mass in mice without CKD (Sato et al., 2022). However, the full impact of these inhibitors on renal osteodystrophy and vascular calcification requires further thorough investigation.

The intricate signaling pathways activated by PTH also extend to osteoblasts, where it triggers the osteoanabolic Gαs/PKA and Gαq/11/PKC pathways. Interestingly, in mouse models featuring Gαq/11 knockout and induced subtotal nephrectomy, coupled with a high dietary phosphate load, there is evidence of reduced bone volume and exacerbated mineral bone disease (Zaloszyc et al., 2022). This highlights the crucial role of PTH signaling in maintaining bone health and the potential consequences of disrupting these pathways, particularly in the context of CKD-MBD.

The intricacies of mineral and bone regulation in the context of CKD necessitate innovative strategies that go beyond conventional treatments.

## Renal osteodystrophy

CKD leads to the development of renal osteodystrophy (ROD), a bone disorder characterized by altered bone mineralization and compromised quality. ROD exhibits manifestations of either high or low bone turnover (Babayev and Nickolas, 2015). In cases of high bone turnover ROD, distinguished by elevated parathyroid hormone (PTH) levels, there is a significant increase in bone turnover involving both formation and resorption processes. Conversely, low bone turnover ROD, linked to suppressed PTH, undergoes reduced mineralization due to decreased osteoblast and osteoclast activity. However, osteoblasts from both conditions in CKD-MBD patients, when isolated from the systemic milieu and cultured, display impaired mineralization associated with compromised maturation (Pereira et al., 2018). Despite efforts to correct serum phosphate and/or PTH levels, mineralization abnormalities persist (Pereira et al., 2009; Pereira et al., 2019). This underscores the pivotal role of internal bone metabolism in the intricate dynamics of ROD.

Several studies have delved into understanding the internal bone mechanisms involved in ROD. These investigations aim to uncover the intricate processes within the bone tissue that influence mineralization, independent of hormonal regulation.

Recent research has shed light on the pivotal role of hepatocyte nuclear factor 4α (HNF4α) in internal bone metabolism within the context of ROD. HNF4α, primarily recognized as a transcription factor expressed in the liver, has been found to be expressed in bone tissue as well. Notably, the expression of HNF4α is diminished in both patients and mice exhibiting ROD. Deletion of Hnf4α specifically in osteoblasts leads to impaired osteogenesis in cells and mice. Conversely, the osteoblast-specific overexpression of *Hnf4α2* has been shown to prevent bone loss in mice with CKD (Martinez-Calle et al., 2023). This underscores the multi-organ involvement in ROD and significance of HNF4α in orchestrating bone health and its potential as a therapeutic target for ROD in CKD.

Another factor influencing internal bone metabolism in CKD is transforming growth factor-β1 (TGF-β1), a well-known regulator of bone turnover. In bone biopsies from individuals with CKD, the expression of TGF-β1 is elevated. The introduction of a neutralizing anti-TGF-β antibody (1D11) in an adenine-induced mouse model of ROD led to a dose-dependent increase in bone volume and a suppression of elevated bone turnover. These effects were associated with reductions in osteoblast and osteoclast surface areas, partially attributed to the inhibition of β-catenin signaling (Liu et al., 2014). The findings highlight the intricate interplay between TGF-β1 and bone metabolism a relationship likely not exclusive to renal osteodystrophy (ROD). Potential complications of TGF-β antibody treatment include off-target effects, given the broad impact of the TGF-β pathway on fibrosis and inflammation. Although there was consideration for TGF-β antibodies to halt the progression of CKD, all trials thus far have yielded negative results, potentially due to blocking the circulating latent form of TGF-β1 (Shi et al., 2011) which has known beneficial effects across multiple systems.

Sclerostin, a soluble antagonist of canonical Wnt/β-catenin signaling and a known inhibitor of bone formation, has also emerged as an important player in mitigating ROD changes. Sclerostin knockout mice subjected to 5/6 nephrectomies and a high phosphate diet for 11 weeks demonstrated reduced ROD changes (Kaesler et al., 2018). Furthermore, studies investigating the administration of anti-sclerostin antibodies in animals with advanced CKD revealed improved bone properties, but notably, this effect was observed only when PTH levels were low (Moe et al., 2015). In animals with high PTH levels, the efficacy of anti-sclerostin antibody was compromised, likely due to PTH suppressing SOST (sclerostin gene) expression (Cejka et al., 2011), interference with Wnt receptor, or the effects of hyperphosphatemia and increased FGF23. Despite the potential promise of anti-sclerostin antibody treatment for ROD with suppressed PTH, improvements in bone histomorphometry did not translate to enhanced bone biomechanical properties (Moe et al., 2015).

The WNT/β-catenin signaling pathway, in which sclerostin plays a role, also interacts with other signaling pathways, such as the bone morphogenetic protein pathway, to collectively regulate CKD-MBD (Zhang et al., 2023). The intricate crosstalk between these pathways underscores the complexity of internal bone mechanisms and offers potential targets for therapeutic interventions in CKD-associated mineral and bone disorders.

## Conclusion

**FIGURE 1:**
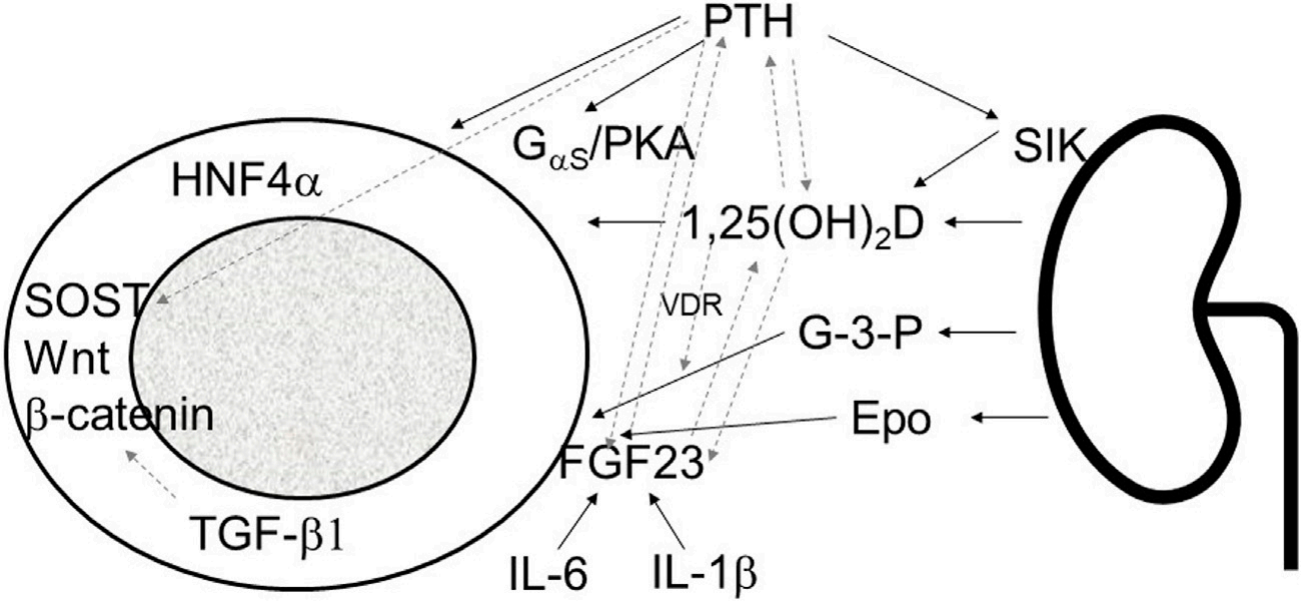
Schematic presentation of kidney bone interaction involving systemic mineral bone factors and intrinsic osseous mechanisms. PTH, parathyroid hormone; SIK sodium inducible kinase; 1,25(OH)_2_D; 1,23 dihydroxy vitamin D; G-3-P, glycerol-3-phosphate; Epo, erythropoietin; FGF23, fibroblast growth factor 23; IL-1β, interleukin 1β; IL-6, interleukin-6; PKA, protein kinase A; HNF4α, hepatocyte nuclear factor 4α; SOST, sclerostin; TGF-β1, transforming growth factor-β1; solid line arrows, pathways described in the manuscript, dashed line arrows, interactions between different pathways.

In conclusion, bone disease in CKD presents a multifaceted challenge involving both systemic mineral imbalances and internal bone disturbances. These dual disorders exert significant impacts on overall physiology, emphasizing the need for a comprehensive approach that addresses both aspects simultaneously. The intricate interplay between systemic factors, such as mineral metabolism regulators like FGF23, PTH, and calcitriol, and intrinsic bone mechanisms, including transcription factors like HNF4α, TGF-β1, and sclerostin, highlights the complexity of CKD-associated MBD (Figure 1).

Despite advancements in our understanding of the underlying mechanisms, there is a conspicuous lack of effective treatments that can comprehensively address the morbidity and mortality associated with CKD-MBD. This underscores the pressing need for continued research efforts to unravel the intricate details of the pathophysiology involved and to identify novel therapeutic targets.
